# Severe Gastrooesophageal Reflux Disease Associated with Foetal Alcohol Syndrome

**DOI:** 10.1155/2012/509253

**Published:** 2012-08-21

**Authors:** N. K. Sujay, Matthew Jones, Emma Whittle, Helen Murphy, Marcus K. H. Auth

**Affiliations:** ^1^Department of Paediatric Gastroenterology, Alder Hey Children's NHS Foundation Trust, Liverpool L12 2AP, UK; ^2^Department of Paediatric General Surgery, Alder Hey Children's NHS Foundation Trust, Liverpool L12 2AP, UK; ^3^Department of Paediatric Dietetics, Alder Hey Children's NHS Foundation Trust, Liverpool L12 2AP, UK; ^4^Department of Clinical Genetics, Liverpool Women's NHS Foundation Trust, Liverpool L8 7SS, UK

## Abstract

Prenatal alcohol exposure may have adverse effects on the developing foetus resulting in significant growth restriction, characteristic craniofacial features, and central nervous system dysfunction. The toxic effects of alcohol on the developing brain are well recognised. However, little is known about the effects of alcohol on the developing gastrointestinal tract or their mechanism. There are few case reports showing an association between foetal alcohol syndrome and gastrointestinal neuropathy. We report a rare association between foetal alcohol syndrome and severe gastrooesophageal reflux disease in an infant who ultimately required fundoplication to optimise her growth and nutrition. The child had failed to respond to maximal medical treatment (domperidone and omeprazole), high calorie feeds, PEG feeding, or total parenteral nutrition. The effect of alcohol on the developing foetus is not limited to the central nervous system but also can have varied and devastating effects on the gastrointestinal tract.

## 1. Case Report

A 3-month-old girl was admitted to hospital for assessment and management of faltering growth. The child was born at 39 weeks' gestation by emergency caesarean section due to foetal distress following induction of labour for intrauterine growth restriction. There were no problems in the immediate postnatal period, and she did not need admission to special care. Birth weight was 2.4 kg (0.4th–2nd centile), and head circumference was 33 cm (9th centile). She was bottle fed, consuming around 4 to 5 oz., 3 to 4 hourly with 4 or 5 bowel movements per day. She showed poor weight gain. Her mother's previous two pregnancies had also resulted in small for gestational age babies (1.4 kg at 34 weeks and 2.3 kg at 41 weeks' gestation). Both family and social history were fully explored during the admission. There was a history of significant alcohol consumption in the mother for which she had undergone a detoxification course during pregnancy.

**Figure 1:**
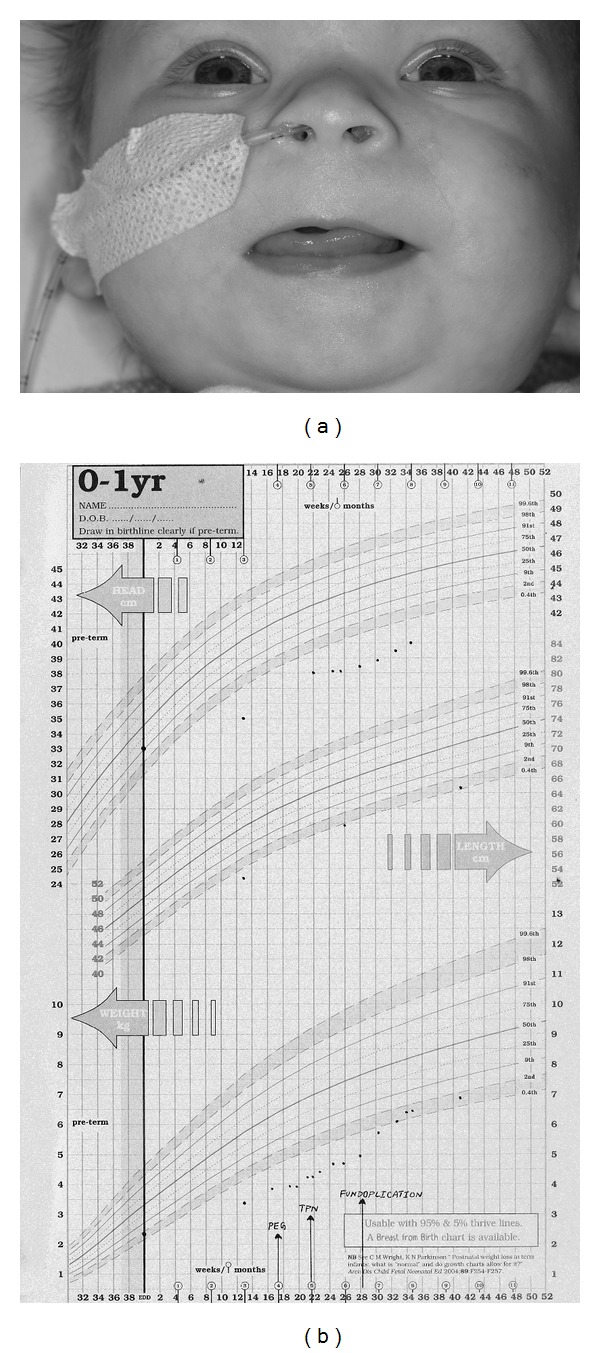
(a) Foetal alcoholic syndrome: face, note the short palpebral fissures, flat nasal bridge and small nose, strikingly featureless philtrum and poorly formed upper lip. (b) Severe malnutrition and growth restriction in foetal alcoholic syndrome. Initially all treatments failed to establish adequate nutrition and growth, including insertion of a gastrostomy and total parenteral nutrition, due to persistent severe vomiting. After 6 months of age, a fundoplication was performed, and gradually enteral nutrition could be reestablished. The child has caught up weight and growth into the percentiles but is still dependent on gastrostomy feeding at present (4 years and 10 months of age).

**Figure 2:**
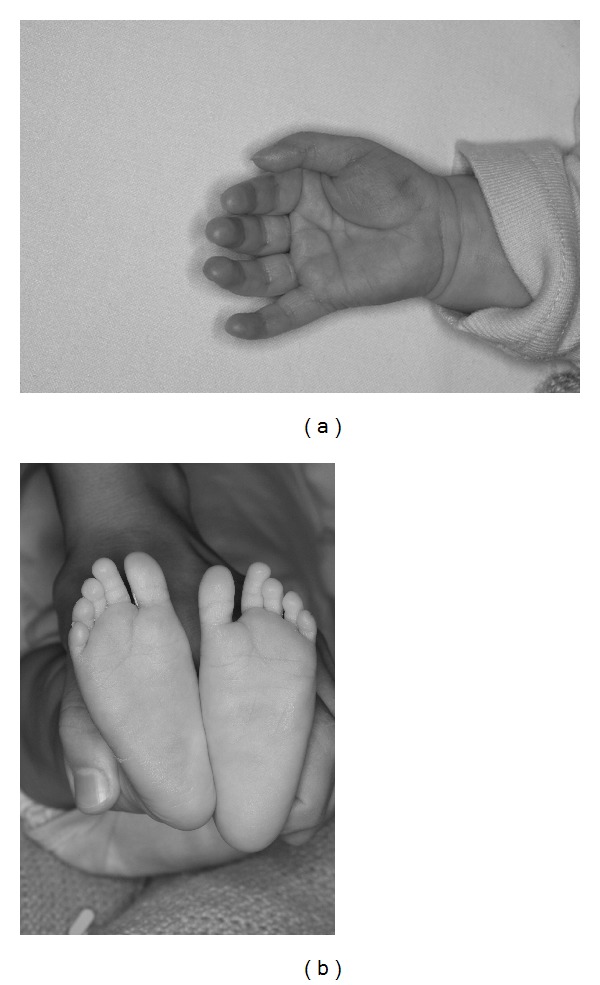
(a) Foetal alcoholic syndrome: right hand, note the “hockey stick” appearance of the deep palmar crease. (b) Feet, note the wide 1-2 sandal gap bilaterally.

On admission the child weighed 3.4 kg with a length of 52.4 cm (both below 0.4th centile). She had microcephaly with a head circumference of 35 cm (less than 0.4th centile). On examination she had several distinctive features with a wide anterior fontanelle, large mouth and tongue, short anteverted nose, flat nasal bridge, long smooth philtrum, thin tented upper lip (Figure 1(a)), short neck, widely spaced nipples, mild camptodactyly of the left fifth finger and deep palmar crease on the right hand (Figure 2(a)), wide sandal gap on both feet (Figure 2(b)), and deep sacral crease. She also had a heart murmur which had been present since birth. A small atrial septal defect was noted on echocardiography. Her growth was falling further away from her centiles (Figure 1(b)), and she was extensively investigated.

The child was reviewed by the genetics team in view of her facial features. Chromosome tests including karyotype and FISH tests for William syndrome and DiGeorge syndrome were all normal. Skin biopsy (for evidence of chromosome mosaicism) and telomere studies were also normal.

She had a normal neurological examination including MRI brain and fundus. CSF analysis was normal including a normal lactate. Full blood count, coagulation profile, biochemistry profile including ammonia and lactate, liver function, bone profile, coeliac screen, inflammatory markers, thyroid function tests, sweat test, and kidney scan were all within normal limits. Metabolic investigations including tests for galactosemia (GALIPUT), fatty acid oxidation disorders, peroxisomal disorders, Smith-Lemli-Opitz syndrome (7-dehydrocholesterol levels), congenital disorders of glycosylation (sialylated transferrin), and maternal phenylketonuria did not reveal any abnormalities.

## 2. Course in the Hospital

The child was taking “SMA Gold” milk feeds via bottle with nasogastric tube top-up feeds to offer nutritional support. She developed RSV-positive bronchiolitis during her stay in hospital and required full nasogastric feeds. Following bronchiolitis she failed to resume oral feeding and became dependent on NG feeds. She also developed a persistent cough which caused significant respiratory distress and vomiting. Her chest X-ray was normal, and it was felt that the cough was secondary to gastrooesophageal reflux. She began to show aversive feeding behaviour, and a PEG tube was inserted at 4 months of age to support her nutrition. However, she continued to vomit and failed to show adequate weight gain even with high calorie formula feeds (Infatrini) via the PEG tube. She was subsequently prescribed hydrolysate milk formula (Nutramigen) and even elemental milk feeds (Neocate), neither of which made a significant difference to her growth or symptoms. A Broviac (central) line was inserted at 5 months of age, and total parenteral nutrition was commenced, but she still showed little weight gain even with high calorie content. She continued to have significant vomiting and had problems tolerating even small amounts of enteral feeds.

She underwent barium studies which showed evidence of gastrooesophageal reflux without any anatomical abnormalities. She also had a 24-hour pH study which again showed evidence of gastrooesophageal reflux. Upper GI endoscopy was normal. Despite maximal medical treatment (domperidone and omeprazole) for gastrooesophageal reflux, she did not show much improvement, and hence she was referred to the surgical team. Fundoplication with vagotomy and pyloroplasty was performed at six and half months of age, after which she started to tolerate enteral feeds via the PEG tube. Parenteral nutrition was weaned off completely over approximately three weeks. Subsequently, she continued to show good progress with gradual and incremental weight gain on full enteral feeds (Infatrini) via PEG. She was discharged from hospital at around 8 months of age, and she is doing well at followup with regard to her growth. She has also started to take solid foods by mouth. Her development is appropriate for age.

## 3. Discussion

In this case, foetal alcohol syndrome was diagnosed based on the characteristic facial features, growth restriction, and central nervous system involvement (microcephaly) along with the history of significant maternal alcohol consumption [1]. She was also reviewed by appropriate specialist teams and investigated for any other diagnosis which could explain all her symptoms. She presented with severe gastrooesophageal reflux which was not responding to medical management and was compromising her growth and nutrition. She required high calorie feeds, PEG insertion, prolonged parenteral nutrition, and eventually fundoplication in order to reverse her growth failure.

The toxic effects of alcohol on the developing brain are well recognised. The most striking abnormalities are those related to impaired neuronal and glial migration [2]. However, relatively little has been reported about the effects of alcohol on the developing gastrointestinal tract or their mechanism. There are case reports suggesting an association between alcohol exposure in pregnancy and gastrointestinal involvement in the form of pyloric stenosis in the neonate [3], enteric neuropathy presenting in infancy as chronic intestinal pseudoobstruction [4] and feeding dysfunction and significant delay in oromotor development [5]. However, there are no reports of foetal alcohol syndrome associated with severe gastrooesophageal reflux disease, as is seen in our case. The purpose of this case report is to highlight the importance of and also raise awareness of the varied gastrointestinal manifestations of infants and children with foetal alcohol syndrome. The exact mechanism by which alcohol affects the gut is not fully understood however we speculate that alcohol can affect the function of the gastrointestinal nervous system and that these effects can be as damaging as those to the foetal brain. Further epidemiological and basic studies are required to explore further the incidence and mechanisms of alcohol-related gastrooesophageal reflux and dysmotility in neonates and infants.
